# Treatment of wild-type mice with 2,3-butanediol, a urinary biomarker of *Fmo5* ^−/−^ mice, decreases plasma cholesterol and epididymal fat deposition

**DOI:** 10.3389/fphys.2022.859681

**Published:** 2022-08-08

**Authors:** Sunil Veeravalli, Dorsa Varshavi, Flora H. Scott, Dorna Varshavi, Frank S. Pullen, Kirill Veselkov, Ian R. Phillips, Jeremy R. Everett, Elizabeth A. Shephard

**Affiliations:** ^1^ Department of Structural and Molecular Biology, University College London, London, United Kingdom; ^2^ Medway Metabonomics Research Group, University of Greenwich, Chatham Maritime, United Kingdom; ^3^ Department of Surgery and Cancer, Faculty of Medicine, Imperial College, London, United Kingdom; ^4^ School of Biological and Chemical Sciences, Queen Mary University of London, London, United Kingdom

**Keywords:** antibiotic, fecal transplant, gut microbiome, metabolite, NMR, stomach, urine, cholesterol

## Abstract

We previously showed that *Fmo5* ^−/−^ mice exhibit a lean phenotype and slower metabolic ageing. Their characteristics include lower plasma glucose and cholesterol, greater glucose tolerance and insulin sensitivity, and a reduction in age-related weight gain and whole-body fat deposition. In this paper, nuclear magnetic resonance (NMR) spectroscopy-based metabolite analyses of the urine of *Fmo5* ^−/−^ and wild-type mice identified two isomers of 2,3-butanediol as discriminating urinary biomarkers of *Fmo5* ^*−/−*^ mice. Antibiotic-treatment of *Fmo5* ^*−/−*^ mice increased plasma cholesterol concentration and substantially reduced urinary excretion of 2,3-butanediol isomers, indicating that the gut microbiome contributed to the lower plasma cholesterol of *Fmo5* ^−/−^ mice, and that 2,3-butanediol is microbially derived. Short- and long-term treatment of wild-type mice with a 2,3-butanediol isomer mix decreased plasma cholesterol and epididymal fat deposition but had no effect on plasma concentrations of glucose or insulin, or on body weight. In the case of long-term treatment, the effects were maintained after withdrawal of 2,3-butanediol. Short-, but not long-term treatment, also decreased plasma concentrations of triglycerides and non-esterified fatty acids. Fecal transplant from *Fmo5* ^*−/−*^ to wild-type mice had no effect on plasma cholesterol, and 2,3-butanediol was not detected in the urine of recipient mice, suggesting that the microbiota of the large intestine was not the source of 2,3-butanediol. However, 2,3-butanediol was detected in the stomach of *Fmo5* ^*−/−*^ mice, which was enriched for *Lactobacillus* genera, known to produce 2,3-butanediol. Our results indicate a microbial contribution to the phenotypic characteristic of *Fmo5* ^*−/−*^ mice of decreased plasma cholesterol and identify 2,3-butanediol as a potential agent for lowering plasma cholesterol.

## Introduction

Flavin-containing monooxygenases (FMOs) are a small family of proteins. Five functional forms, FMOs 1, 2, 3, 4 and 5, are present in both human and mouse. An additional four, apparently functional, *Fmo* genes are present in mice, *Fmo6*, *9*, *12*, and *13* (Hernandez et al., 2004); however, the functional capacities of their protein products have not been established. FMOs catalyze the oxygenation of a wide range of substrates, which include pharmaceuticals and environmental chemicals, leading to the excretion of the oxygenated product (Krueger and Williams, 2005; Phillips and Shephard, 2017). FMOs are therefore generally considered as detoxification enzymes and have recently been shown to play a role in stress resistance (Huang et al., 2021).

One member of the FMO family, FMO5, is unusual in that it catalyzes Baeyer-Villiger monooxygenation of several foreign chemicals (Fiorentini et al., 2016; Fiorentini et al., 2017). The ability of FMO5, but not other FMOs, to carry out this type of catalysis has been explained recently by a comparison of crystal structures of ancestral FMOs (Nicoll et al., 2020).

The creation of an *Fmo5* ^*−/−*^ mouse has identified FMO5 as an enzyme that is involved also in endogenous metabolic processes, acting as a regulator of metabolic ageing (Gonzalez Malagon et al., 2015). *Fmo5* ^*−/−*^ mice exhibit an age-related lean phenotype; in comparison with wild-type mice, they have lower plasma concentrations of glucose and cholesterol, greater glucose tolerance and insulin sensitivity, lower inflammatory tone, less whole-body fat deposition and, from about 20-weeks of age, gain less weight (Gonzalez Malagon et al., 2015; Scott et al., 2017). In addition, FMO5 has been implicated as a sensor of gut bacteria (Scott et al., 2017).

Nuclear magnetic resonance (NMR) spectroscopy-based analyses of urine of *Fmo5* ^−/−^ and wild-type mice identified age-related differences in a number of urinary metabolites (Varshavi et al., 2018). We now report the identification of two isomers of 2,3-butanediol that are discriminating urinary biomarkers of *Fmo5* ^*−/−*^ mice. Antibiotic treatment of *Fmo5* ^*−/−*^ mice increased plasma cholesterol concentrations and substantially reduced urinary excretion of 2,3-butanediol, showing that gut bacteria contribute to the lower plasma cholesterol of *Fmo5* ^−/−^ mice, and that 2,3-butanediol is microbially derived. We hypothesised that treatment of wildtype mice with 2,3-butanediol would lower cholesterol and potentially also lower epididymal fat. We report here that treatment of wild-type mice with 2,3-butanediol does prevent age-related increases in the plasma concentration of cholesterol and reduces the deposition of epididymal fat.

## Methods

### Animal maintenance and sample collection

*Fmo5* ^*−/−*^ mice, generated on a C57BL/6J background (Gonzalez Malagon et al., 2015), and wild-type C57BL/6J mice were bred at University College London and housed with free access to food and water, as described previously (Gonzalez Malagon et al., 2015). Urine, blood and tissue samples were collected between 9:00 a.m. and 12 noon, as described (Gonzalez Malagon et al., 2015; Veeravalli et al., 2017). All procedures were carried out under appropriate Home Office Licences in accordance with the UK Animal Scientific Procedures Act and with local ethics committee approval (Animal Welfare and Ethical Review Body). Details of animal numbers, timing, doses etc are provided in the Figure captions.

### Analysis of urine and stomach contents

Stomach contents were prepared for metabolite analysis as previously described (Varshavi et al., 2020). All NMR experiments and data analyses were conducted at 600 MHz for ^1^H NMR as previously described (Varshavi et al., 2018), including data acquisition and processing.

Briefly, for urine, all samples were collected onto an ice-cooled surface and then frozen on solid CO_2_ and stored at 193 K until transported on solid CO_2_ for analysis. 50 μl of thawed urine from each mouse was mixed with 25 μl of phosphate buffer (81/19 (v/v) 0.6 M K_2_HPO_4_/0.6 M NaH_2_PO_4_ in 100% ^2^H_2_O, pH 7.4, containing 0.5 mM sodium 3-(trimethylsilyl)-2,2′,3,3′-tetradeuteropropionate (TSP), as NMR shift reference, and 9 mM sodium azide, as an anti-microbial. Buffered samples were centrifuged at 13,000 g for 5 min at 4°C to remove any suspended particles. 60 μl of supernatant was then transferred into new 1.7 mm outer diameter NMR tubes (Norell, S-1.7-500-1) using an accurate, long-needle electronic syringe (SGE eVol XR). Samples were loaded into 96-well plates in a Bruker SampleJet sample changer (Bruker BioSpin, Germany) where the temperature was maintained at 277 K. Each sample was automatically pre-heated to 300.0 K prior to data acquisition.

^1^H NMR spectra were recorded on a Bruker Avance III spectrometer (Bruker BioSpin GmbH, Rheinstetten, Germany) operating at 600.44 MHz and at a temperature of 300.0 K. A standard one-dimensional (1D) NOESY presaturation pulse sequence, Bruker code noesygppr1d, with gradient pulses (RD-90°-*t*1-90°-*t*m-90°-acquire) was acquired with water suppression applied during the relaxation delay of 2 s, with a mixing time of 100 ms and a 90° pulse of 11.2 μs. For each spectrum, 8 dummy scans were used to establish spin-state equilibrium, then 256 free induction decay transients were collected into 65,536 data points with a spectral width of 20 ppm.

NMR spectra were processed using the software TopSpin 3.2 (Bruker BioSpin, UK). Prior to applying Fourier transformation, free induction decays were multiplied by an exponential function corresponding to a line broadening of 0.3 Hz. The 1D ^1^H NMR spectra were manually phased, baseline corrected and referenced to the chemical shift of TSP (0.0 ppm).

2D NMR methods such as J-resolved, correlation spectroscopy (COSY), total correlation spectroscopy (TOCSY), HSQC and HMBC were acquired to assist with spectral interpretation and metabolite identification. Representative experimental data acquisition and data processing parameters for all these experiments on the urine of a week 60 FMO5 knockout mouse are given in Supplementary Table S1. The key experiments for metabolite identification were 2D ^1^H, ^1^H COSY, 2D ^1^H, ^13^C HSQC and 2D ^1^H, ^13^C HMBC experiments which enabled proton-to-proton correlations over 2 to 6 bonds, proton to carbon-13 correlations over 1 bond and proton to carbon-13 correlations over 2 to 3 bonds respectively, to be determined.

Metabolite identification was accomplished according to published guidelines (Sumner et al., 2007; Everett, 2015; Dona et al., 2016). The 1D and 2D NMR spectra shown in the Figures were processed using MNova 11.0 (Mestrelab Research S.L.). Metabolites were quantified by NMR signal area measurements as described previously (Veeravalli et al., 2020).

### Identification of isomers of 2,3-butanediol in urine of *Fmo5* ^*−/−*^ mice

Urine was spiked with authentic meso-2,3-butanediol and 2R,3R-butanediol (Sigma-Aldrich), and analysed by NMR, as described above. High-resolution ultra-performance liquid chromatography–mass spectrometry (UPLC-MS) was conducted on urine samples from 30-week-old *Fmo5* ^*−/−*^ and wild-type mice, essentially as described previously (Varshavi et al., 2018), with the following modifications. Urine samples (60 μl) were centrifuged (13,000g for 10 min at 4°C) to remove particulates. Supernatant (50 μl) was diluted with 100 μl of water, centrifuged at 13,000g for 5 min at 4°C then transferred into a vial before injection into the UPLC-TOF-MS (Waters, Manchester, UK). UPLC was in isocratic mode with an eluent of 0.1% formic acid, 10% acetonitrile. The flow rate was 0.4 ml/min, injection volume was 1μl, the MS source temperature was 130°C and the scan time was 0.5 s. To attempt to distinguish between the two enantiomeric forms of 2,3-butanediol, urine was spiked with the enantiomeric NMR shift reagents sodium [(R)-1,2-diaminopropane-*N*,*N*,*N′*,*N′*-tetraacetato] samarate (III), hydrate and sodium [(S)-1,2-diaminopropane-*N*,*N*,*N′*,*N′*-tetraacetato] samarate (III), hydrate (Tokyo Chemical Industry UK Ltd.) and analysed by 600 MHz ^1^H NMR.

### Antibiotic treatment

Mice were treated by oral dosing of ampicillin (1 g/l) and neomycin (0.5 g/l) in their drinking water for 14 days. Antibiotic-containing water was replenished twice weekly, and animals were given free access to the standard chow diet, as described previously (Scott et al., 2017).

### 2,3-butanediol treatment

Wild-type mice were dosed orally in their drinking water with 2,3-butanediol in a 4:1:1 ratio of meso-2,3-butanediol (Sigma-Aldrich):2R,3R-butanediol (Alfa-Aesar):2S,3S-butanediol (Fischer Scientific). Dosing regimes and animal numbers are given in the text and figure legends.

### Plasma analyses

Plasma was isolated from freshly collected blood samples, as described (Hough et al., 2002), and biochemical analyses carried out at the Medical Research Council Harwell Institute (Harwell, Oxfordshire, UK). Insulin was measured by enzyme-linked immunosorbent assay, as described previously (Scott et al., 2017).

### Fecal transplant

Fresh fecal lysates were prepared, as described (Willing et al., 2011), from donor *Fmo5* ^*−/−*^ male mice (*n* = 3), which were 16-weeks old at the start of the experiment. The recipient group were wild-type, male mice treated with a single injection of streptomycin (2 mg/g body weight) 1 day before the first fecal transplant dose (Willing et al., 2011). Recipient mice were treated with fecal lysate via oral gavage on days 1, 4, and 7. An age-matched control group was treated via oral gavage with phosphate-buffered saline on days 1, 4, and 7. At the start of the experiment recipient and control wild-type mice were 9-weeks old. Urine was collected 1 day after each gavage treatment and then at 13, 15, and 18 weeks of age. Blood was collected at 18 weeks of age.

### Intestinal microbiota analysis

Stomach contents were isolated after dissection of the intestinal tract. Stomach bacteria were identified and quantified, via analysis of 16S ribosomal RNA gene sequences, as described previously (Scott et al., 2017).

### Statistical analyses

Data are expressed as means ± SEM. Statistical significance was determined using an unpaired two-tailed *t* test and GraphPad Prism (version 9.1.0; GraphPad Software, Inc., La Jolla, CA) or by false-discovery-rate (FDR)-controlled ANOVA as described previously (Varshavi et al., 2018).

## Results

### Isomers of 2, 3-butanediol are present in the urine of *Fmo5* ^*−/−*^ mice, but not in that of wild-type mice

**FIGURE 1:**
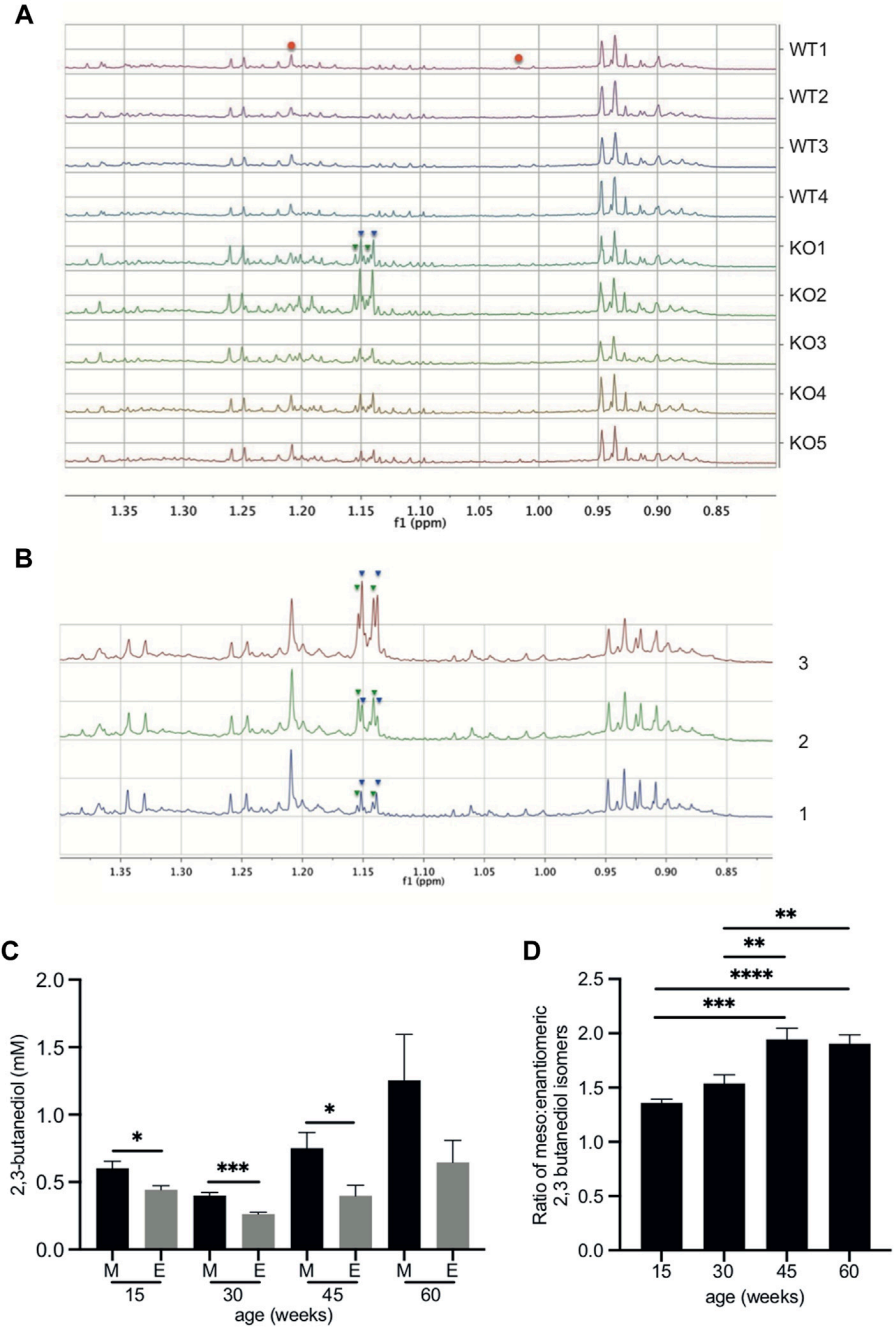
2,3-butanediol is present in the urine of *Fmo5* ^*−/−*^ but not wild-type mice. **(A)** The low-frequency region of the 600 MHz ^1^H NMR spectra from 60-week-old male *Fmo5* ^*−/−*^ (KO) and wild-type (WT) mice. The pseudo-doublet signals at ca. 1.146 (inverted blue triangles) and 1.149 ppm (inverted green triangles) are present only in the spectra of the urine of *Fmo5* ^*−/−*^ mice. Note that the singlet at ca. 1.208 ppm and the broad triplet at ca. 1.015 ppm are due to methyl signals from 6-hydroxy-6-methyl-heptan-3-one (red circles), and are at a lower intensity in older mice, as this male sex pheromone has been shown to decrease with age (Varshavi et al., 2018). **(B)** Confirmation of 2,3-butanediol identity in *Fmo5* ^*−/−*^ mouse urine. (1) The low-frequency region of the 600 MHz ^1^H NMR spectrum of urine from a 30-week-old male *Fmo5* ^*−/−*^ mouse. (2) The NMR spectrum of the same urine sample rerun after the addition of a small quantity of authentic 2R, 3R-butanediol (inverted green triangles) into the urine. (3) The NMR spectrum after a subsequent addition of authentic meso-2,3-butanediol (inverted blue triangles) into the urine. **(C)** Concentrations of 2,3 butanediol isomers in *Fmo5* ^−/−^ male mouse urine. Meso (M) and enantiomeric (E) forms were quantified in urine from mice aged 15 (*n* = 4 KO, *n* = 4 WT), 30 (*n* = 4 KO, *n* = 4 WT; set 1: *n* = 5 KO, *n* = 5 WT; set 2), 45 (*n* = 5 KO, *n* = 4 WT) and 60 (*n* = 5 KO, *n* = 4 WT) weeks. **(D)** Ratio of meso: enantiomeric forms of 2,3-butanediol in *Fmo5* ^−/−^ male mouse urine at different ages. Data in parts **(C,D)** are expressed as means ± SEM. *p < 0.05, **p < 0.01, ***p < 0.001, ****p < 0.0001.

Previously we have reported an NMR-based analysis comparing the urinary metabolite profiles of *Fmo5* ^*−/−*^ and wild-type male mice as they age (Varshavi et al., 2018). Here we extend this study to investigate discriminating features between *Fmo5* ^*−/−*^ and wild-type mouse urine. Unbiased, unsupervised, multivariate principal component analysis of the ^1^H NMR spectra of urine from *Fmo5* ^*−/−*^ mice showed it to be distinct from the urine of age-matched wild-type mice at all timepoints studied: 15, 30, 45 and 60 weeks of age. Unusual methyl proton signals at ca. 1.146 and 1.149 ppm (Figure 1A and Supplementary Figure S1) were present in the ^1^H NMR spectra of urine of all *Fmo5* ^*−/−*^ mice and *absent* from the spectra of urine of all wild-type mice; at 60-weeks of age (Figure 1A, *n* = 5 KO, *n* = 4 WT, *p* = 0.00015 and *p* = 0.0002) and also at 15-weeks (Supplementary Figure S1, *n* = 4 KO, *n* = 4 WT, *p* = 0.0002 and *p* = 0.0003), 30-weeks (*n* = 4 KO, *n* = 4 WT, *p* = 0.000008 and *p* = 0.0002), 30-weeks (Set 2 repeat study, *n* = 5 KO, *n* = 5 WT, *p* = 0.000004 and *p* = 0.000002) and 45-weeks of age (*n* = 5 KO, *n* = 4 WT, *p* = 0.00006 and *p* = 0.00001, all values for the signals at ca. 1.146 and 1.149 ppm respectively and all false discovery rate controlled with an FDR = 0.1).

The metabolites responsible for the unique signals in *Fmo5* ^*−/−*^ mouse urine were identified by a series of 2D NMR spectra. A 2D ^1^H J-resolved NMR spectrum (Supplementary Figure S2) showed that the pseudo-doublets (Figure 1A) were in fact complex, second order, multiplet signals, with the signal at 1.146 ppm having at least six transitions and that at 1.149 ppm having at least four transitions, which is unusual for methyl group resonances. A 2D ^1^H COSY (correlated spectroscopy) NMR spectrum of *Fmo5* ^*−/−*^ mouse urine (Supplementary Figure S3) showed clear vicinal coupling connectivities from the methyl signals at 1.146 and 1.149 ppm to methyne hydrogens, resonating at ca. 3.734 and 3.630 ppm respectively. These data indicated that the signals were due to the meso- (Human Metabolite Database, HMDB03156) and enantiomeric (HMDB33007) isomers of the small diol, 2,3-butanediol. The identity of the metabolites was confirmed by 2D ^1^H HSQC (heteronuclear single quantum coherence) and HMBC (heteronuclear multiple bond correlation) NMR spectra, with spectral data matching those of reference materials (Supplementary Table S2). These identifications were verified by spiking authentic reference standards into the urine sample of an *Fmo5* ^*−/−*^ mouse (Figure 1B).

Orthogonal confirmation of the identification of 2,3-butanediol was achieved by high-resolution UPLC electrospray MS experiments. Authentic meso- and 2R,3R-butanediol eluted at retention times of ca. 1.0 and 1.1 min respectively. The positive-ion electrospray mass spectra of the two isomers were dominated by [M + H-H_2_O]^+^ peaks at m/z 73.0646 (theoretical 73.0648, taking into account the mass of the electron; mass error 2.7 ppm). The single-ion chromatogram for m/z 73.0650 showed peaks at 1.0 and 1.1 min in urine from a 30-week-old *Fmo5* ^*−/−*^ male mouse, confirming the presence of both the meso- and enantiomeric-isomers of 2,3-butanediol. No such signals were present in the urine of a 30-week-old wild-type mouse, confirming the absence of 2,3-butanediol isomers.

To determine which isomer or isomers of enantiomeric-2,3-butanediol were present, experiments were conducted with both the R and S enantiomers of the aqueous-soluble, chiral chemical shift reagent [1,2-diaminopropane-*N*,*N*,*N′*,*N′*-tetraacetato] samarate (III). Unfortunately, no significant enantiomer-specific chemical shift changes were observed in the ^1^H NMR spectra when these reagents were added to authentic 2R,3R- and 2S,3S-butanediol. Thus, the chirality of the enantiomeric 2,3-butanediol observed in the urine of *Fmo5* ^*−/−*^ mice was not determined.

### Ratio of 2,3-butanediol isomers in urine of *Fmo5* ^*−/−*^ mice

Quantification of the NMR signals for the 2,3-butanediol isomers in the urine of 15-, 30-, 45- and 60-week-old male *Fmo5* ^*−/−*^ mice showed that the meso form was always present in a greater amount than the enantiomeric form(s) (Figure 1C) and the ratio of meso to enantiomeric form(s) increased with age (Figure 1D).

### Antibiotic treatment of *Fmo5* ^*−/−*^ mice increases plasma cholesterol concentration and decreases the urinary concentration of 2,3-butanediol

**FIGURE 2:**
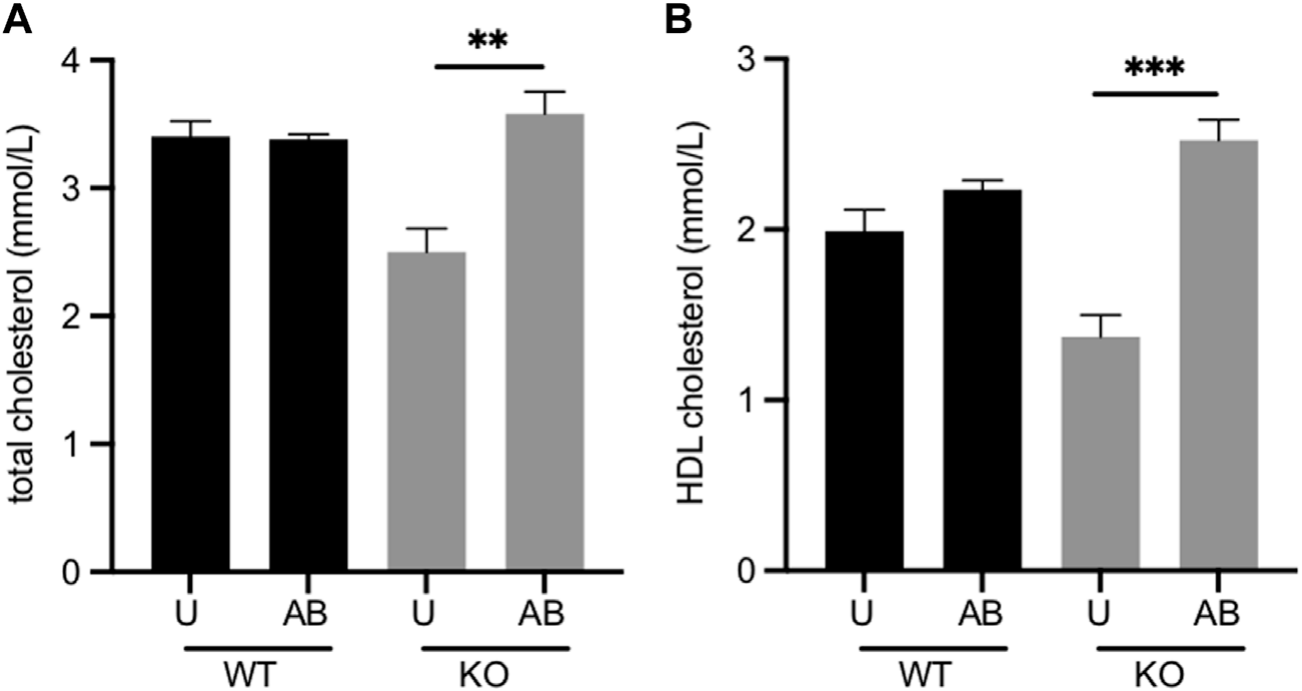
Effect of antibiotic treatment on plasma concentrations of total cholesterol. **(A)** and HDL cholesterol. **(B)** Male *Fmo5* ^*−/−*^ (KO) or wild-type (WT) mice (30-weeks old) were treated with antibiotics in their drinking water for 2 weeks (AB) or were untreated (U). WT, *n* = 4; WT + AB, *n* = 4; KO, *n* = 4; KO + AB, *n* = 5. Data are expressed as means ± SEM. **p < 0.01, ***p < 0.001.

*Fmo5* ^*−/−*^ mice have lower plasma concentrations of total and HDL cholesterol than their wild-type counterparts (Gonzalez Malagon et al., 2015). The gut microbiota is known to influence cholesterol metabolism of the host animal (Kriaa et al., 2019). To determine whether the gut microbiota contributed to the lower plasma cholesterol of *Fmo5* ^*−/−*^ mice we investigated the effect of antibiotic treatment. Treatment of *Fmo5* ^*−/−*^ mice with antibiotics increased the plasma concentrations of both total cholesterol, from 2.50 ± 0.18 mmol/l to 3.58 ± 0.17 mmol/l (*p* < 0.01), and HDL cholesterol, from 1.37 ± 0.13 mmol/l to 2.52 ± 0.12 mmol/l (*p* < 0.001), in each case reaching levels similar to those in wild-type mice (Figures 2A,B). In contrast, antibiotic treatment of wild-type mice had no effect on plasma concentrations of either total cholesterol or HDL cholesterol (Figures 2A,B). The results indicate that the lower plasma cholesterol concentration of *Fmo5* ^*−/−*^ mice was influenced by the gut microbiome.

To determine whether the 2,3-butanediol detected in *Fmo5* ^*−/−*^ mouse urine was of microbial origin, urine from antibiotic-treated *Fmo5* ^*−/−*^ mice was analysed by ^1^H NMR. Antibiotic treatment decreased the concentrations of the meso and enantiomeric isomers by about 85% and 80% respectively. The number of bacteria detected in feces of antibiotic-treated *Fmo5* ^*−/−*^ and wild-type mice was substantially depleted and the small population that remained was dominated by Firmicutes (94%) in both sets of animals. Although 2,3-butanediol was not fully eliminated by the antibiotic regime used, the results indicate that both the meso and enantiomeric forms are largely of microbial origin.

### Treatment of wild-type mice with 2,3-butanediol prevents an age-related increase in plasma cholesterol concentration

Based on the finding that antibiotic treatment of *Fmo5* ^*−/−*^ mice increased plasma concentrations of total and HDL cholesterol, and concomitantly reduced the concentrations of 2,3-butanediol, we hypothesized that 2,3-butanediol affects plasma cholesterol concentration. To test this, we investigated the effect of 2,3-butanediol treatment of wild-type mice on their plasma cholesterol concentration. Quantification of 2,3-butanediol isomers in the urine of *Fmo5* ^*−/−*^ mice (Figure 1D) indicated that a reasonable approximation for dosing wild-type mice would be a 2:1 mix of the meso:enantiomeric forms. Because the proportion of the R,R and S,S enantiomers of 2,3-butanediol in *Fmo5* ^*−/−*^ mouse urine is not known, we used a 4:1:1 mix of meso:R,R:S,S isomers. We aimed to dose wild-type mice with an amount that would result in their excreting 2,3-butanediol in urine in concentrations similar to those excreted by *Fmo5* ^*−/−*^ mice. To determine an appropriate dose, wild-type mice were treated with a range of concentrations of the 4:1:1 mix of 2,3-butanediol isomers in their drinking water and the 2,3-butanediol excreted in urine quantified. The dose received by each animal was calculated on the basis of the average daily water intake of the mice. Mice dosed with the equivalent of 250 mg/kg/d had a urinary concentration of 2,3-butanediol in the range excreted by *Fmo5* ^*−/−*^ mice.

**FIGURE 3:**
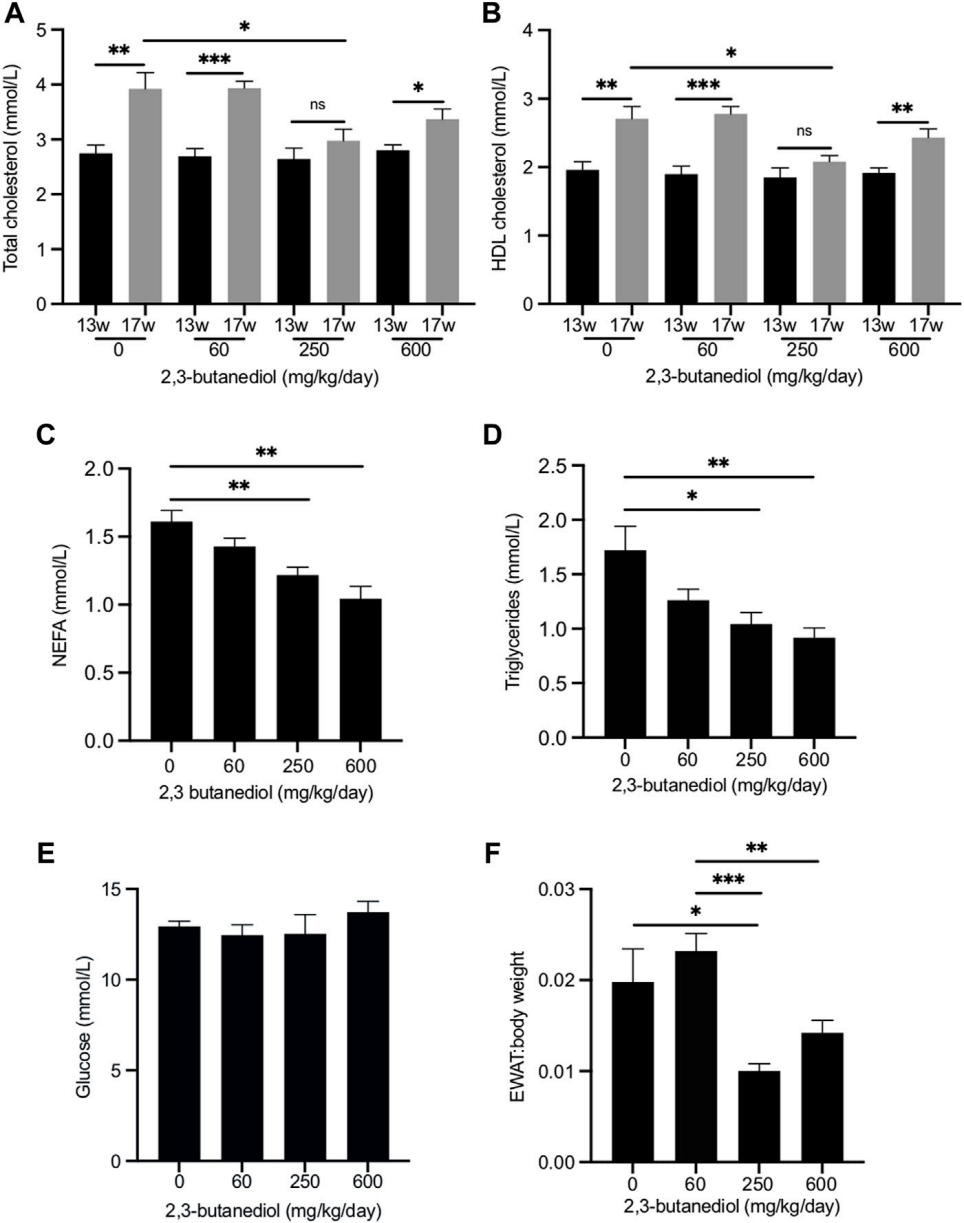
Dose-dependent influence of 2,3-butanediol treatment on plasma metabolite concentrations and fat deposition. **(A)** Total cholesterol. **(B)** HDL cholesterol. **(C)** NEFA. **(D)** Triglycerides. **(E)** Glucose **(F)** EWAT:body weight ratio. Male mice were either untreated (0 mg/kg/day) or treated with 2,3-butanediol at either 60, 250 or 600 mg/kg/day, for 4 weeks from week 13 (13w) to week 17 (17w) of age. Cohorts, *n* = 4 or 5. Data are expressed as means ± SEM. *p < 0.05, **p < 0.01, ***p < 0.001.

Wild-type mice, aged 13 weeks, were randomly placed into four cohorts and their plasma cholesterol measured. There was no significant difference in plasma concentrations of either total or HDL cholesterol among the four cohorts (Figures 3A,B). Cohorts were then treated with 2,3-butanediol for 4 weeks at doses of 60, 250 or 600 mg/kg/d or were untreated. 2,3-butanediol was present in the urine of all treated mice, but was absent from urine of untreated animals (Supplementary Table S3). No differences were detected by ANOVA, with an FDR of 0.1, in the abundance of any other metabolites in the urine of treated and untreated mice. However, taurine levels were statistically significantly, inversely correlated with 2,3-butanediol levels in the urines of the treated male mice (data not shown).

Over the 4-week period, from age 13–17 weeks, in untreated mice the plasma concentration of total cholesterol increased from 2.75 ± 0.15 mmol/l to 3.92 ± 0.30 mmol/l (*p* < 0.01), an increase of 43% (Figure 3A), and that of HDL cholesterol from 1.96 ± 0.11 mmol/l to 2.71 ± 0.18 mmol/l (*p* < 0.01), an increase of 38% (Figure 3B). Similar age-related increases in plasma total cholesterol and HDL cholesterol have been reported (Gonzalez Malagon et al., 2015). After 4 weeks of treatment with 2,3-butanediol at a dose of 250 mg/kg/d, plasma concentrations of both total cholesterol (2.98 ± 0.21 mmol/l) and HDL cholesterol (2.08 ± 0.09 mmol/l) were significantly lower (*p* < 0.05) than those of mice that were untreated for 4 weeks, and not significantly different from the concentrations at the start of the treatment (Figures 3A,B). Therefore, 2,3-butanediol, at a dose of 250 mg/kg/d, prevented age-related increases in plasma concentrations of total and HDL cholesterol. However, treatment of mice with 2,3-butanediol at doses of 60 or 600 mg/kg/d did not prevent age-related increases in plasma concentrations of either total or HDL cholesterol, which reached levels that were not significantly different from those of mice untreated for 4 weeks (Figures 3A,B).

### 2,3-butanediol treatment decreases the plasma concentrations of non-esterified fatty acids and triglycerides

We also investigated the effect of 2,3-butanediol treatment on the plasma concentrations of non-esterified fatty acids (NEFA) and triglycerides. At the start of the experiment (13-week-old mice) there were no significant differences in NEFA or triglyceride concentrations among the four cohorts. After 4 weeks, NEFA and triglyceride concentrations of untreated mice were not significantly different from those at the start of the experiment. Treatment of mice for 4 weeks with 2,3-butanediol at a dose of either 250 or 600 mg/kg/d significantly decreased plasma concentrations of both NEFA and triglycerides, whereas a dose of 60 mg/kg/d had no significant effect (Figures 3C,D).

### 2,3-butanediol treatment has no effect on plasma concentrations of glucose or insulin

2,3-butanediol treatment at doses of 60, 250 or 600 ng/kg/d had no effect on the plasma concentration of glucose (Figure 3E). After treatment of mice with 250 mg/kg/d, the plasma concentration of insulin (1.93 ± 0.51 ng/ml) was not significantly different from that of untreated mice (1.75 ± 0.19 ng/ml). The effect on plasma insulin concentration of doses of 60 and 600 mg/kg/d was not determined.

### 2,3-butanediol treatment decreases the ratio of epididymal white adipose tissue to body weight

Treatment of mice with 2,3-butanediol had no effect on food intake or body weight. However, the EWAT:body weight ratio was significantly decreased in mice dosed with 2,3-butanediol at 250 mg/kg/d (Figure 3F). Although the EWAT:body weight ratio was lower following a 2,3-butanediol dose of 600 mg/kg/d, it was not significantly different from that of untreated mice (Figure 3F).

### Long-term treatment with 2,3-butanediol prevents an age-related increase in plasma cholesterol concentration and decreases the EWAT:body weight ratio

Having established that a relatively short-term (4-week) treatment with 2,3-butanediol prevented an age-related increase in plasma cholesterol and decreased the EWAT:body weight ratio, we investigated whether these effects could be maintained or enhanced over a longer-term treatment and whether they could be sustained after withdrawal of 2,3-butanediol. Eight-week-old male wild-type mice were treated with the same 4:1:1 mix of meso:R,R:S,S 2,3-butanediol isomers at a dose of 200 mg/kg/d. At 22 weeks of age, that is, after 14 weeks of treatment, 2,3-butanediol was withdrawn from the drinking water of half the animals, which were maintained for a further 6 weeks on water with no added 2,3-butanediol. This cohort was termed “washout”. Analysis of urine of the washout cohort, collected 1 week after withdrawal of treatment, confirmed the absence of 2,3-butanediol. The remaining animals continued to be treated with 2,3-butanediol for a further 6 weeks. This cohort was termed “throughout”. Both cohorts were aged 28 weeks at the end of the experiment.

**FIGURE 4:**
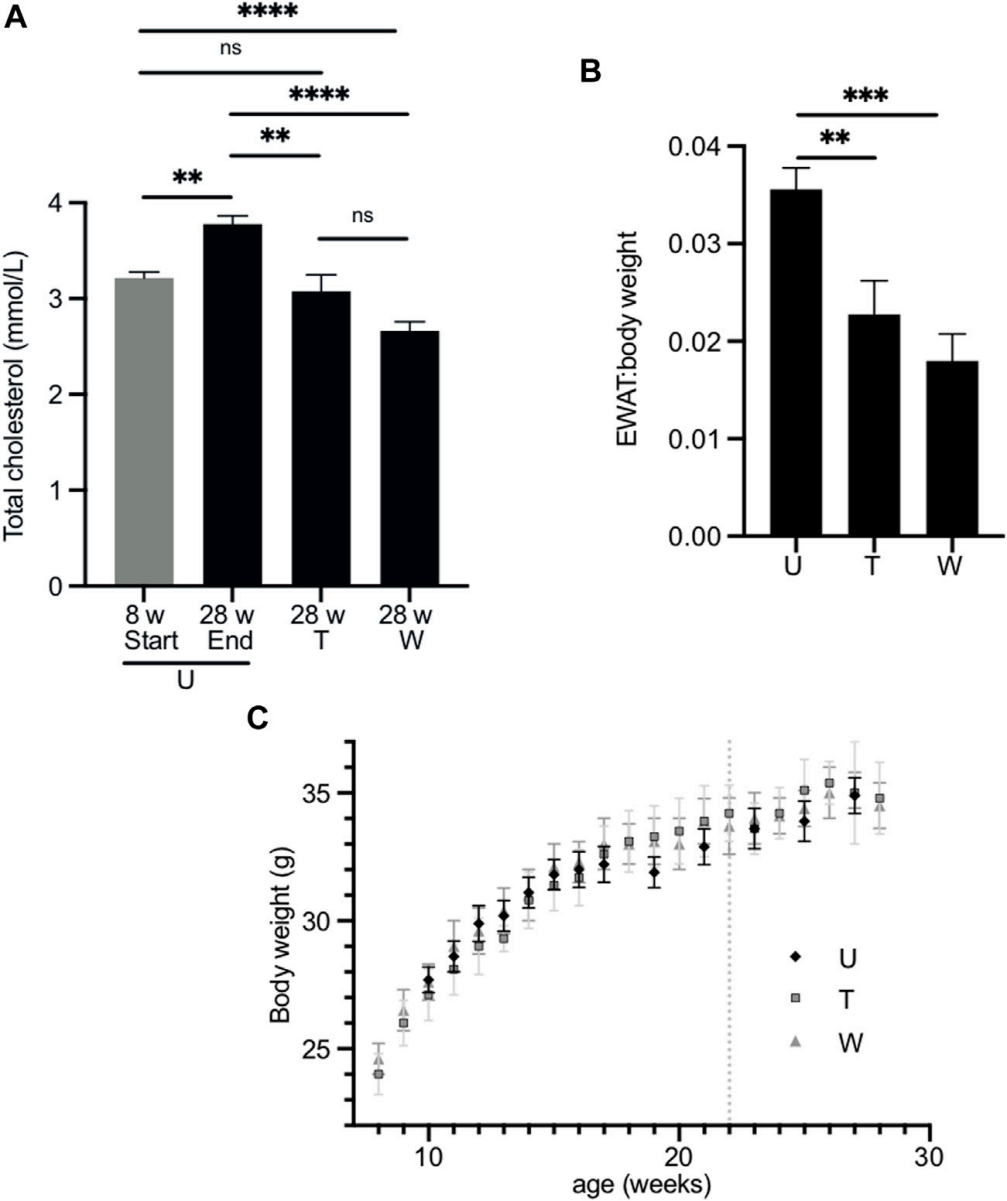
Effect of long-term treatment with 2,3-butanediol on plasma cholesterol, EWAT:body weight ratio and body weight. **(A)**. Male mice were untreated (U) and aged 8 (*n* = 8) or 28 weeks of age (*n* = 23). The “washout” cohort (W) (*n* = 4) were treated with 2,3-butanediol for 14 weeks (from 8 to 22 weeks of age) and then maintained on water to 28 weeks of age. The “throughout” cohort (T) (*n* = 4) were treated with 2,3-butanediol from 8 to 28 weeks of age. **(B)** EWAT:body weight ratio of U, W or T mice at 28 weeks of age. **(C)** Body weight over time of U, W, and T cohorts. Dotted line indicates withdrawal of 2,3-butanediol from the washout cohort. Data are expressed as means ± SEM. **p < 0.01, ***p < 0.001, ****p < 0.0001.

At 28 weeks, the plasma total cholesterol concentrations of the throughout (3.07 ± 0.18 mmol/l) and washout (2.67 ± 0.09 mmol/l) cohorts were significantly lower than that of age-matched untreated mice (3.78 ± 0.09 mmol/l) (Figure 4A) and were similar to those of age-matched *Fmo5* ^*−/−*^ mice (2.88 ± 0.12 mmol/l) (Gonzalez Malagon et al., 2015). The plasma cholesterol concentration of the throughout cohort was similar to that of eight-week-old mice at the start of the experiment (3.22 ± 0.06 mmol/l); however, that of the washout cohort was lower (Figure 4A). In contrast, the plasma cholesterol concentration of 28-week-old untreated mice was significantly higher than that of eight-week-old mice (Figure 4A).

At the end of the treatment regime, the body weight and weight of EWAT was determined for each of the cohorts. The EWAT:body weight ratios of both the throughout (0.023 ± 0.003) and washout (0.018 ± 0.003) cohorts were significantly less than that of age-matched, untreated mice (0.036 ± 0.002) (Figure 4B) and were similar to that of age-matched *Fmo5* ^*−/−*^ mice (Gonzalez Malagon et al., 2015). Therefore, as with short-term treatment, long-term treatment with 2,3-butanediol prevented an age-related increase in plasma cholesterol concentration and decreased the EWAT:body weight ratio, and both effects were sustained after withdrawal of 2,3-butanediol, i.e., in the throughout and washout treatment regimes. However, in contrast to short-term treatment with 2,3-butanediol, long-term treatment had no effect on plasma concentrations of NEFA or triglygerides.

During the course of the treatment, from eight- to 28-weeks of age, the body weights of both the throughout and washout male cohorts were similar to those of age-matched untreated animals (Figure 4C), as was their food intake (data not shown). This is in contrast to *Fmo5* ^*−/−*^ mice, which exhibit a reduction in weight gain from about 20-weeks of age, and by 30-weeks of age weigh ∼10% less than wild-type mice, despite greater intake of food (Gonzalez Malagon et al., 2015). As was the case with short-term treatment, long-term treatment with 2,3-butanediol had no effect on the plasma concentrations of glucose or insulin.

In treated animals, the plasma concentrations of alkaline phosphatase, alanine aminotransferase, aspartate aminotransferase, creatine kinase and lactate dehydrogenase were within the normal range for these enzymes (Hough et al., 2002), indicating that long-term treatment of mice with 2,3-butanediol had no overt deleterious effects on organ or tissue function.

### Fecal transplant from *Fmo5* ^*−/−*^ mice to wild-type mice has no effect on plasma cholesterol concentration or fat deposition

By 20 weeks of age, in comparison with wild-type mice, male *Fmo5* ^*−/−*^ mice have a lower plasma cholesterol concentration and begin to exhibit a reduction in body weight gain (Gonzalez Malagon et al., 2015). The composition of the gut microbiota of *Fmo5* ^*−/−*^ mice differs from that of wild-type mice and the differences become more pronounced as the mice age (Scott et al., 2017). To explore the possibility that gut bacteria present in *Fmo5* ^*−/−*^ mice could affect plasma cholesterol concentration or body weight gain, we carried out fecal transplants from *Fmo5* ^*−/−*^ mice to wild-type mice.

**FIGURE 5:**
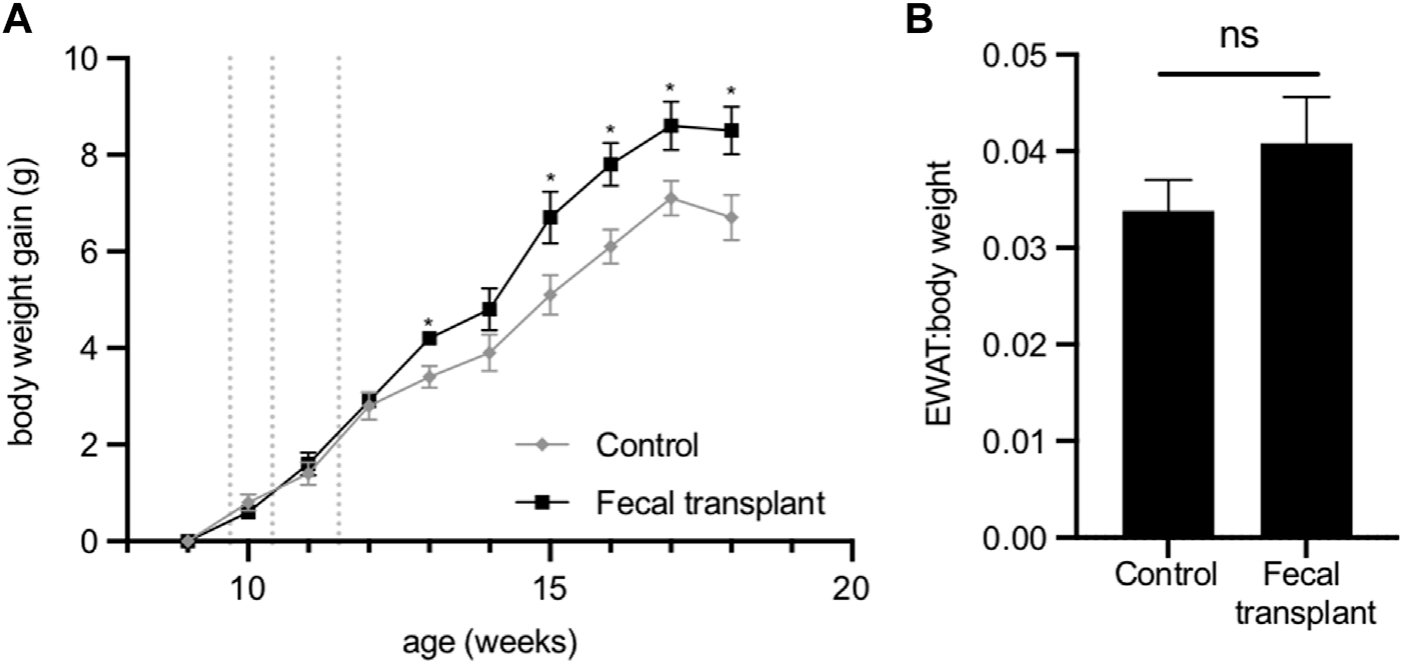
Effect of fecal transplantation of wild-type mice with Fmo5−/− gut bacteria. **(A)** Body weight gain. Dotted lines indicate the days of dosing of fecal material. *p < 0.05. **(B)** EWAT:body weight ratio of mice at 18 weeks of age. Control (*n* = 5) and fecal-transplanted mice (*n* = 5).

No differences were observed in the plasma concentrations of cholesterol, HDL cholesterol, triglycerides, NEFA, glucose, ketone bodies or insulin between the transplanted mice and control animals (Supplementary Table S4). Analysis of urine of the transplanted mice detected no 2,3-butanediol. After fecal transplantation from *Fmo5* ^*−/−*^ mice, wild-type mice surprisingly gained significantly more weight than age-matched non-transplanted mice (Figure 5A). The increased gain in body weight of the transplanted mice was accompanied by an increase in EWAT:body weight ratio, but this was not statistically significant (Figure 5B).

### The composition of the stomach microbiota of *Fmo5* ^*−/−*^ and wild-type mice is different

The results of the fecal transplant suggested that bacteria present in the large intestine of *Fmo5* ^*−/−*^ mice do not affect plasma cholesterol concentration, but, unexpectedly, appear to be involved in promoting weight gain. This raised the question of the location of bacteria that produce 2,3-butanediol, and the possibility that 2,3-butanediol is produced by bacteria higher up the digestive tract, in either the stomach or small intestine.

**FIGURE 6:**
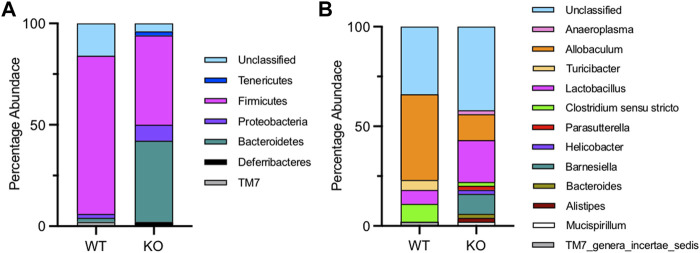
Stomach microbial phyla **(A)** and genera **(B)** of wild-type (WT) and *Fmo5* ^*−/−*^ mice (KO).

Figure 6 shows the composition of the microbiota in the stomach of wild-type and *Fmo5* ^*−/−*^ mice. In wild-type mice, the stomach microbiota was dominated by the phylum Firmicutes (77%), with Bacteroidetes representing only 2%, whereas in *Fmo5* ^*−/−*^ mice, the phyla Firmicutes and Bacteroidetes were present in almost equal abundance (Firmicutes 44%, Bacteroidetes 40%), and there was a higher proportion of Proteobacteria in *Fmo5* ^*−/−*^ mice (8%) than in wild-type mice (2%) (Figure 6A). At the genus level, *Allobaculum* (phylum, Firmicutes) dominates in wild-type mice (43%), whereas in *Fmo5* ^*−/−*^ mice the dominant genus was *Lactobacillus* (phylum, Firmicutes), at 21% (Figure 6B). The genus *Lactobacillus* is known to produce 2,3-butanediol (Ledingham and Neish, 1954) and NMR analysis detected signals close to those expected for both isomers of the metabolite at low levels in the stomach contents of *Fmo5* ^*−/−*^ mice (Supplementary Figure S3), but not in the contents of duodenum, jejunum, ileum, cecum or colon.

## Discussion

Our results identify meso and enantiomeric isomers of 2,3-butanediol as urinary biomarkers of *Fmo5* ^*−/−*^ mice. Studies in rats and humans demonstrate that 2,3-butanediol can be produced endogenously, via a secondary pathway of ethanol metabolism (Rutstein et al., 1983; Otsuka et al., 1996; Otsuka et al., 1999). The substantial decrease in the urinary concentration of 2,3-butanediol as a consequence of antibiotic treatment of *Fmo5* ^*−/−*^ mice supports a microbial origin of the 2,3-butanediol. However, we cannot rule out the possibility that the endogenous pathway contributes to the production of 2,3-butanediol.

In bacteria, the meso and enantiomeric isomers of 2,3-butanediol are produced from pyruvate as the end products of sugar fermentation (Ledingham and Neish, 1954; Zhang et al., 2016). The production of 2,3-butanediol, which is pH neutral, is considered a survival pathway to counteract lethal acidification generated by low-pH fermentation products (Bartowsky and Henschke, 2004; Lee et al., 2015). Of the two enantiomeric isomers of 2,3-butanediol, bacteria predominantly produce the 2R,3R form and production of 2S,3S form is uncommon (Ledingham and Neish, 1954). However, as we were unable to differentiate between the two isomers, we cannot be certain that the enantiomeric isomer we detect in the urine of *Fmo5* ^*−/−*^ mice is exclusively the 2R,3R form.

Short-term treatment of wild-type mice with a mixture of meso- and enantiomeric-2,3-butanediol isomers prevented age-related increases in the plasma concentrations of total and HDL cholesterol and decreased the EWAT:body weight ratio, both of which are phenotypic characteristics of *Fmo5* ^*−/−*^ mice (Gonzalez Malagon et al., 2015). However, 2,3-butanediol treatment of wild-type mice had no effect on other phenotypic characteristics of *Fmo5* ^*−/−*^ mice, namely, reduced weight gain and lower plasma concentrations of glucose and insulin (Gonzalez Malagon et al., 2015; Scott et al., 2017). Our results indicate that the lower plasma cholesterol concentration of *Fmo5* ^*−/−*^ mice is profoundly influenced by the gut microbiome. In contrast, the reduced weight gain and decreased plasma concentrations of glucose and insulin of *Fmo5* ^*−/−*^ mice are independent of the gut microbiota (Scott et al., 2017). The ability of 2,3-butanediol to elicit in wild-type mice metabolic phenotypes characteristic of *Fmo5* ^*−/−*^ mice is therefore limited to those that are influenced by the gut microbiota, which is consistent with the microbial origin of 2,3-butanediol.

The decrease in the plasma concentrations of NEFA and triglycerides in response to short-term treatment of wild-type mice with 2,3-butanediol demonstrates that it can produce metabolic effects not observed in *Fmo5* ^*−/−*^ mice (Gonzalez Malagon et al., 2015). But these effects were not maintained by longer-term treatment with 2,3-butanediol. The basis for the differential effect of short- and long-term treatment on plasma concentrations of NEFA and triglycerides is not known. The effect may be age-related, with younger mice being more responsive than older mice, short- and long-term-treated mice being 17 and 28 weeks of age, respectively, when analysed. Interestingly, it has been reported that 2,3-butanediol is a potent inhibitor of metabolism in rat adipocytes (Lomeo et al., 1988).

The increased rate of body weight gain of wild-type mice as a consequence of fecal transplantation from *Fmo5* ^*−/−*^ mice is unexpected. However, the inability of the fecal transplant to induce, in wild-type mice, the lean phenotypic characteristics of *Fmo5* ^*−/−*^ mice, lean appearance, reduced weight gain and decreased subcutaneous and visceral fat deposits (Gonzalez Malagon et al., 2015), is consistent with the lean phenotype being independent of the fecal microbiota (Scott et al., 2017). In addition, fecal transplantation from *Fmo5* ^*−/−*^ to wild-type mice did not confer the ability to produce 2,3-butanediol and had no effect on plasma cholesterol concentration. It seems likely, therefore, that the source of 2,3-butanediol in *Fmo5* ^*−/−*^ mice is microbes that inhabit the stomach or upper small intestine, not the cecum or colon. Such microbes would be expected to be absent or present in very low amounts in fecal material.

The composition of the stomach microbiota of 30-week-old *Fmo5* ^*−/−*^ and wild-type mice differed markedly (Figure 6), at the levels of both phyla and genera, and the differences were much greater than those between the fecal microbiota of the mice (Scott et al., 2017). For instance, in stomach microbiota the ratio of Firmicutes to Bacteroidetes was 1.1 in *Fmo5* ^*−/−*^ mice and 38 in wild-type mice, whereas in fecal microbiota the ratio was 0.7 and 1.0 respectively (Scott et al., 2017). In *Fmo5* ^*−/−*^ mice, the stomach microbiota is enriched with the genus *Lactobacillus*. In addition to being a potential source of 2,3-butanediol (Ledingham and Neish, 1954), strains of *Lactobacillus* have been shown to have cholesterol-lowering properties, although the mechanisms by which they achieve this are not fully understood (Kriaa et al., 2019). Our data conclusively show that 2,3-butanediol lowers the concentration of plasma cholesterol.

It is not known whether 2,3-butanediol acts directly to generate the lower plasma cholesterol concentration characteristic of *Fmo5* ^*−/−*^ mice. Although direct effects are possible, 2,3-butanediol might exert its influence indirectly by changing the abundance of gut bacterial species that would influence plasma cholesterol concentration, for instance, by increasing the abundance of species that lower plasma cholesterol, such as *Lactobacillus*, or by decreasing the abundance of species that increase plasma cholesterol. Other studies have found links between gut bacteria and plasma cholesterol. For example, *Apoe* ^*−/−*^ mice transplanted with gut microbiota from humans with elevated plasma cholesterol developed a high cholesterol phenotype (Le Roy et al., 2019).

FMO5 is expressed throughout the intestinal tract in cells at the luminal surface (Scott et al., 2017) and we now show that in its absence, in *Fmo5* ^*−/−*^ mice, there is a pronounced effect on the microbial populations present in the stomach. FMO5 can act as an antioxidant and/or detoxifying enzyme and is capable of catalyzing Baeyer-Villiger oxidation reactions on a number of food additives and dietary products (Fiorentini et al., 2016). The enzyme can also act as an NADPH oxidase, producing H_2_O_2_ (Fiorentini et al., 2017). Generation of H_2_O_2_ by FMO5 might influence the habitat of microbes throughout the gut in a manner similar to that proposed for NOX1-derived H_2_O_2_ in the colon (Miller et al., 2020). Although the specific role that FMO5 carries out in the gut lining is not understood, our previous (Scott et al., 2017) and current results (Figure 6) demonstrate that FMO5 plays an important role in influencing the microbial composition of the gut, possibly through modulation of the gut environment.

## Conclusions

In the current study, we show that the gut microbiome contributes to the lower plasma cholesterol concentration of *Fmo5* ^*−/−*^ mice, demonstrating the importance of genome-microbiome interactions in influencing host metabolic phenotypes. Treatment of wild-type mice with 2,3-butanediol, a microbial metabolite which we identified as a urinary biomarker of *Fmo5* ^*−/−*^ mice, prevented an age-related increase in plasma cholesterol concentration, and decreased epididymal fat deposition. Our results identify 2,3-butanediol as a potential therapeutic agent for lowering plasma cholesterol. It has the advantage that its effect is sustained after withdrawal of treatment. A weakness of our study is that the mechanism of cholesterol lowering, and fat deposition reduction needs further investigation, but this may in turn yield new molecular targets for these important therapeutic areas. The effect of lowering HDL cholesterol in addition to total cholesterol may be less desirable if that translates to humans, but the finding that long-term treatment of mice with 2,3-butanediol had no harmful effects, together with its presence in some foods (Buttery et al., 1994; Chung, 1999; Bartowsky and Henschke, 2004), indicate that the use of 2,3-butanediol should be relatively safe.

## Data Availability

The datasets presented in this study can be found in online repositories. The names of the repositories and accession numbers can be found below: Metabolites: Metabolights (EBI) MTBLS4266; 16SrRNA: GenBank (NCBI), ON059716-ON059748 and ON059926-ON059967.

## Abbreviations

EWAT, epididymal white adipose tissue; FMO, flavin-containing monooxygenase; HDL, high density lipoprotein; MS, mass spectrometry; NEFA, non-esterified fatty acid; NMR, nuclear magnetic resonance; UPLC, ultra-performance liquid chromatography.
