# Supplementary material for: Treatment of wild-type mice with 2,3-butanediol, a urinary biomarker of Fmo5 −/− mice, decreases plasma cholesterol and epididymal fat deposition
Source: Front Physiol. 2022 Aug 8;13:859681. doi: 10.3389/fphys.2022.859681 (PMC9393927; doi:10.3389/fphys.2022.859681)
Supplement: Supplementary file 1 [file DataSheet2.pdf]

Supplementary Information

Treatment of wild-type mice with 2,3-butanediol, a urinary biomarker of *Fmo5*$^{-/-}$ mice, decreases plasma cholesterol and epididymal fat deposition

Sunil Veeravalli[1], Dorsa Varshavi[2,5], Flora H. Scott[1], Dorna Varshavi[2], Frank S. Pullen[2#], Kirill Veselkov[3], Ian R. Phillips[1,4] Jeremy R. Everett[2*] and Elizabeth A. Shephard[1*]

[1]Department of Structural and Molecular Biology,
University College London,
London,
United Kingdom, WC1E 6BT

[2]Medway Metabonomics Research Group,
University of Greenwich,
Chatham Maritime,
Kent,
United Kingdom, ME4 4TB

[3]Department of Surgery and Cancer
Faculty of Medicine
Imperial College
London
United Kingdom, SW7 2AZ

[4]School of Biological and Chemical Sciences,
Queen Mary University of London,
London,
United Kingdom, EN4 1NS

[5]Current address
Faculty of Science, Biological Sciences
University of Alberta
Edmonton,
Canada, T6G 2R3

[#] deceased 2021

*corresponding authors
Jeremy R Everett: j.r.everett@greenwich.ac.uk
Elizabeth A Shephard: e.shephard@ucl.ac.uk

Supplementary Figures and Tables

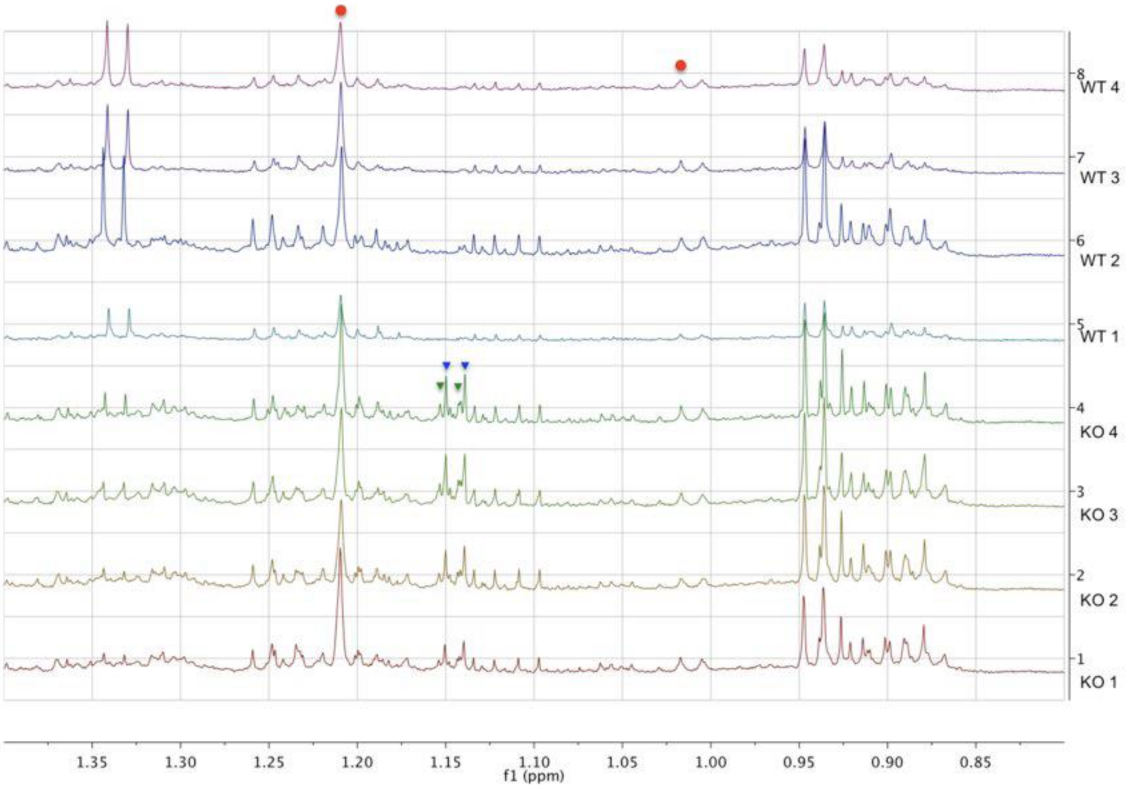

Supplementary Figure 1. The low-frequency region of the 600 MHz ^{1}H NMR spectra of urine from *Fmo5*$^{-/-}$ (KO) and wild-type (WT) male mice. The pseudo-doublet signals at ca. 1.146 (inverted blue triangles) and 1.149 ppm (inverted green triangles) are present only in the spectra of *Fmo5*$^{-/-}$mouse urine. The large, broad singlet at ca. 1.208 ppm and the broad triplet at ca. 1.015 ppm are due to methyl signals from the male sex pheromone 6-hydroxy-6-methyl-heptan-3-one (red circles) (Varshavi et al., 2018). Mice were aged 15 weeks. KO, n=4; WT, n=4.

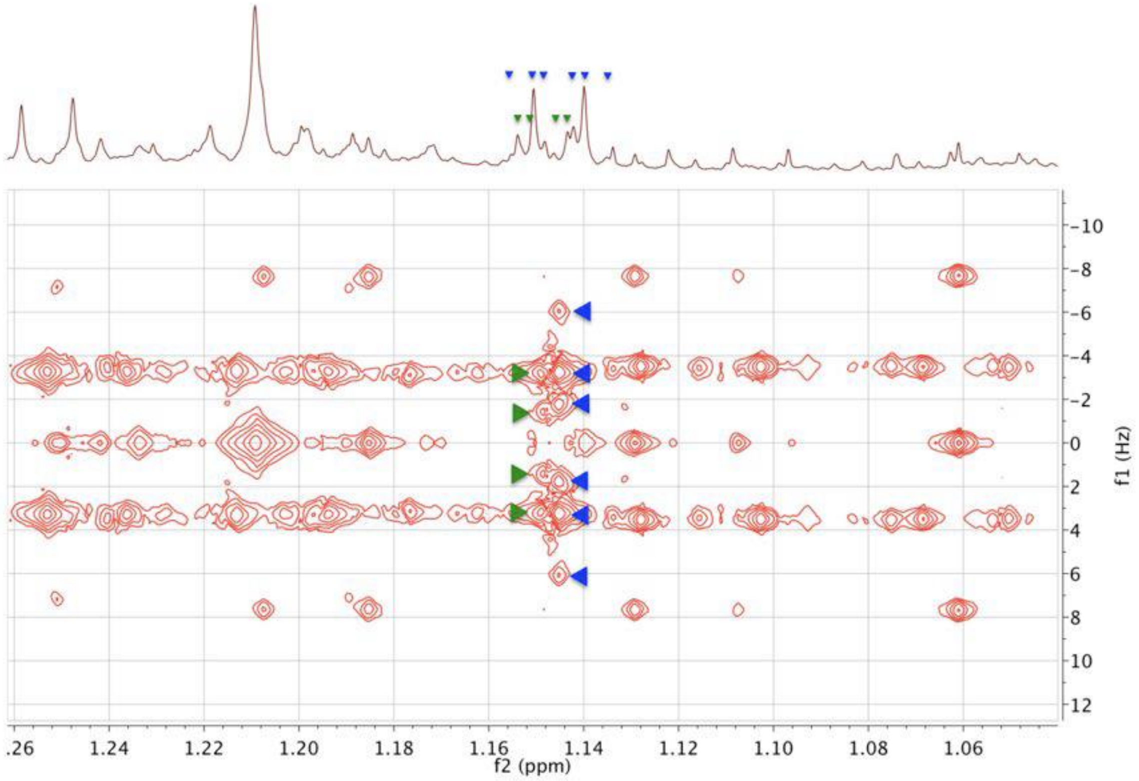

Supplementary Figure 2. The low-frequency region of the 600 MHz 2D ^{1}H J-resolved NMR spectrum of *Fmo5$^{-/-}$* mouse urine. The 2D spectrum is plotted underneath the corresponding 1D ^{1}H NMR spectrum. The x-axis shows chemical shifts and the y-axis shows homonuclear proton couplings. The pseudo-doublet signals at ca. 1.146 (blue triangles) and 1.149 ppm (green triangles) are shown to possess at least 6 and 4 transitions respectively, due to their second order nature. The spectra were from an analysis of urine from a 30-week-old male *Fmo5$^{-/-}$* mouse.

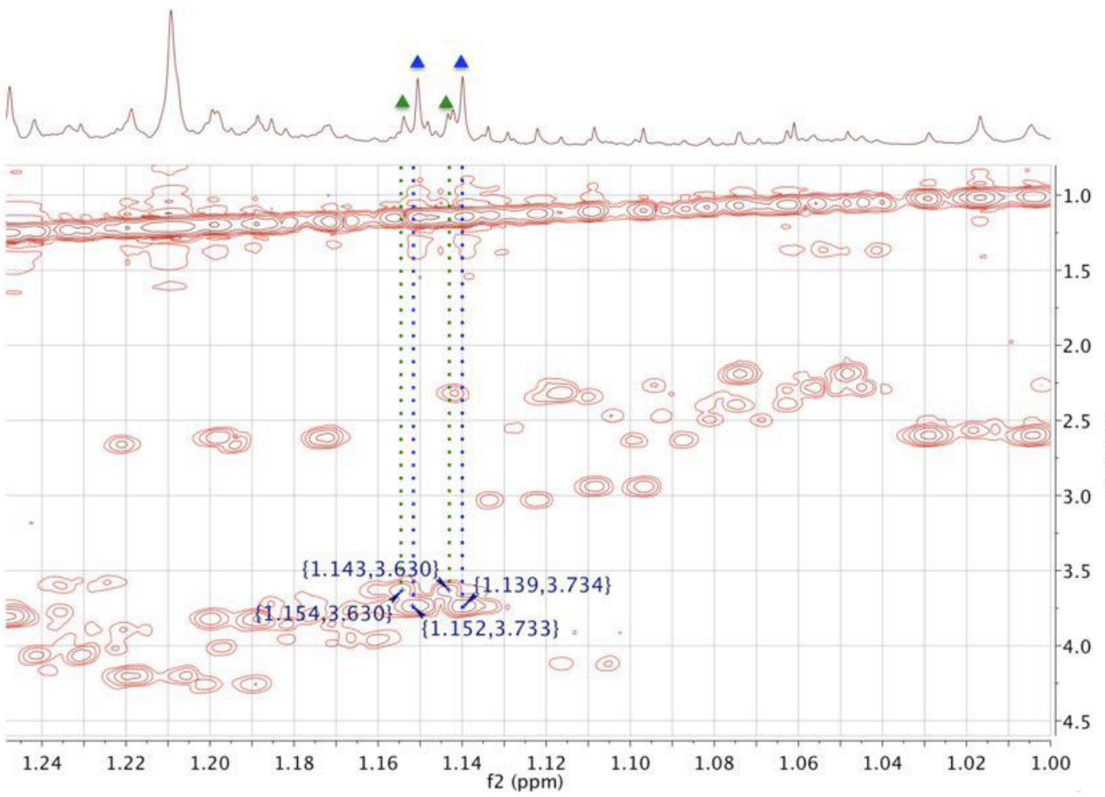

Supplementary Figure 3. The low-frequency region of the 600 MHz 2D ^{1}H COSY NMR spectrum of *Fmo5*$^{-/-}$ mouse urine. The spectrum is plotted underneath the corresponding 1D ^{1}H NMR spectrum. The pseudo-doublet methyl signals at ca. 1.146 (blue triangles) and 1.149 ppm (green triangles) are shown to correlate with methyne signals at ca. 3.734 and 3.630 ppm respectively. The spectra were from an analysis of urine from a 30-week-old male *Fmo5*$^{-/-}$ mouse.

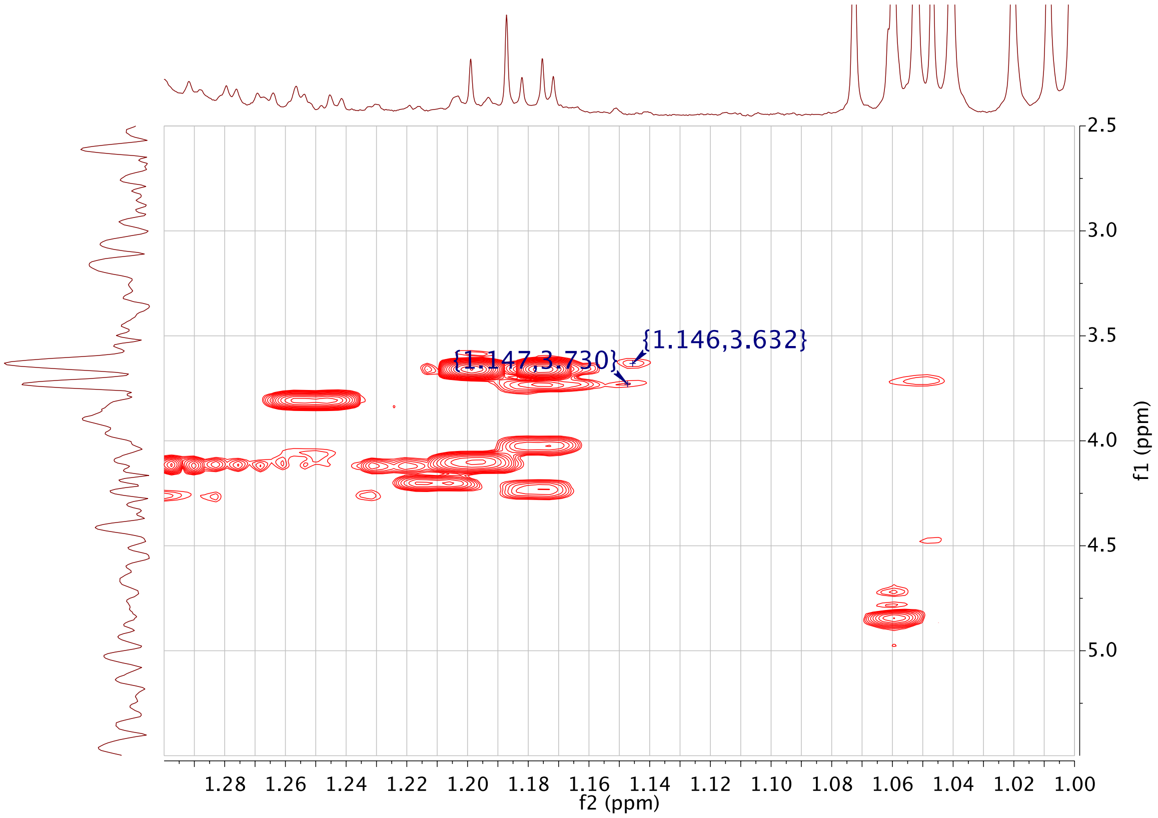

Supplementary Figure 4. An expansion of the low-frequency region of the 600 MHz 2D ^{1}H COSY NMR spectrum of the stomach contents of an *Fmo5$^{-/-}$* mouse. The spectrum is plotted underneath the corresponding 1D ^{1}H NMR spectrum. Weak cross-peaks from methyl signals at ca. 1.146 and 1.147 ppm are shown to correlate with methyne signals at ca. 3.632 and 3.730 ppm respectively and are close in position to those expected for the enantiomeric and meso isomers of 2,3-butanediol.

Supplementary Table 1: data acquisition and processing parameters for 2D NMR spectra of urine samples from an FMO5 knockout mouse at week 60

Parameter	JRES	COSY	TOCSY	HSQC	HMBC
F2 spectral width in Hz	10.026.7	9,578.5	9,590.8	9,615	6,203
F1 spectral width in Hz	78.0	9,578.5	9,596.9	24,875.6	33,523
data points in t2	4,096	4,096	4,096	1,024	2,048
spectral size in F2	8,192	8,192	8,192	4,096	8,192
increments in t1	40	512	600	256	128
spectral size in F1	256	4,096	1,024	2,048	512
number of scans	2	32	32	64	128
relaxation delay (s)	2.00	2.00	2.00	1.50	1.50
apodisation	sine bell in t1 and t2 with first point correction in t1	sine bell in t2, sine bell and first point correction in t1	sine square in t2, sine square and first point correction in t1	sine bell squared in t2, sine square with first point correction in t1	sine bell in t1 and t2 with first point correction in t1
Bruker NMR pulse sequence code	jresgpprqf	cosygpqfpr	mlevgpphprzf.be	hsqcetgpprsisp 2.2	hmbcgplpndqf
notes	tilted and symmetrised	t1 noise reduced		phase-sensitive and multiplicity-edited	

Supplementary Table 2. NMR data for the authentic reference standards of 2,3-butanediol (2,3BD) and data from urine of a 30-week-old male *Fmo5*$^{-/-}$ mouse

sample	δ_H	δ_C	COSY	HMBC
authentic meso-2,3BD	1.143	19.3	3.738	
authentic meso-2,3BD	3.738	73.7	1.143	
authentic 2R,3R-2,3BD	1.147	20.5	3.627	
authentic 2R,3R-2,3BD	3.627	74.2	1.147	
meso-2,3BD in urine of a *Fmo5*$^{-/-}$ mouse	1.146	19.6	3.73	73.6
enantiomeric-2,3BD in urine of a *Fmo5*$^{-/-}$ mouse	1.149	20.7	3.63	74.5

δ_C values are from HSQC spectra. All proton signals in both isomers of 2,3BD are 2nd order multiplets. HMBC indicates long-range ^{13}C to ^{1}H connectivity found in HMBC spectra of mouse urine. All reference standard data are for samples in pH 7.4 deuterated phosphate buffer with TSP reference. Urine samples were prepared as previously described (Varshavi et al., 2018).

Supplementary Table 3. Quantification of 2,3 butanediol in urine of wild-type mice treated with different doses of the molecule.

**Cohort**	**2,3 butanediol (mM)**	**Creatinine (mM)**	**Concentration of 2,3 butanediol normalized to creatinine**
Untreated	0.00	3.12	0.00
Untreated	0.00	1,65	0.00
Untreated	0.00	3.32	0.00
Untreated	0.00	2.15	0.00
60 mg/kg/d	0.39	2.10	0.19
60 mg/kg/d	0.27	0.55	0.50
60 mg/kg/d	0.55	2.66	0.21
60 mg/kg/d	0.39	0.73	0.53
60 mg/kg/d	0.58	2.39	0.24
250 mg/kg/d	4.41	1.93	2.28
250 mg/kg/d	1.58	1.35	1.17
250 mg/kg/d	0.61	1.17	0.52
250 mg/kg/d	1.48	1.27	3.94
250 mg/kg/d	0.81	0.78	1.05
600 mg/kg/d	1.93	0.65	2.95
600 mg/kg/d	1.57	2.05	0.77
600 mg/kg/d	5.00	1.13	4.44
600 mg/kg/d	3.99	2.90	1.38
600 mg/kg/d	4.61	3.58	1.29

2,3 butanediol and creatinine were quantified as described in the methods section. Values of 2,3 butanediol are given relative to creatinine to account for any differences in urine concentration at the time of sample collection.

Supplementary Table 4. Analysis of plasma metabolites of wild-type mice after fecal transplantation from *Fmo5*$^{-/-}$ mice

Cohort	Total cholesterol (mmol/L)	HDL cholesterol (mmol/L)	Glucose (mmol/L)	Triglycerides (mmol/L)	NEFA (mmol/L)	Ketone bodies (mmol/L)	Insulin (ng/mL)
Control (n = 5)	4.3 ± 0.10	3.1 ± 0.06	14.8 ± 0.40	1.48 ± 0.12	1.7 ± 0.10	n.d.	2.1 ± 0.39
Fecal transplant (n = 5)	4.4 ± 0.21	3.1 ± 0.13	15.1 ± 0.17	1.52 ± 0.20	1.6 ± 0.48	n.d	1.9 ± 0.26

Data are means ± SEM. n.d. = not detected.
