# Supplementary material for: Treatment of wild-type mice with 2,3-butanediol, a urinary biomarker of Fmo5 −/− mice, decreases plasma cholesterol and epididymal fat deposition
Source: Front Physiol. 2022 Aug 8;13:859681. doi: 10.3389/fphys.2022.859681 (PMC9393927; doi:10.3389/fphys.2022.859681)

genome – microbiome – metabolome – phenotype linkage

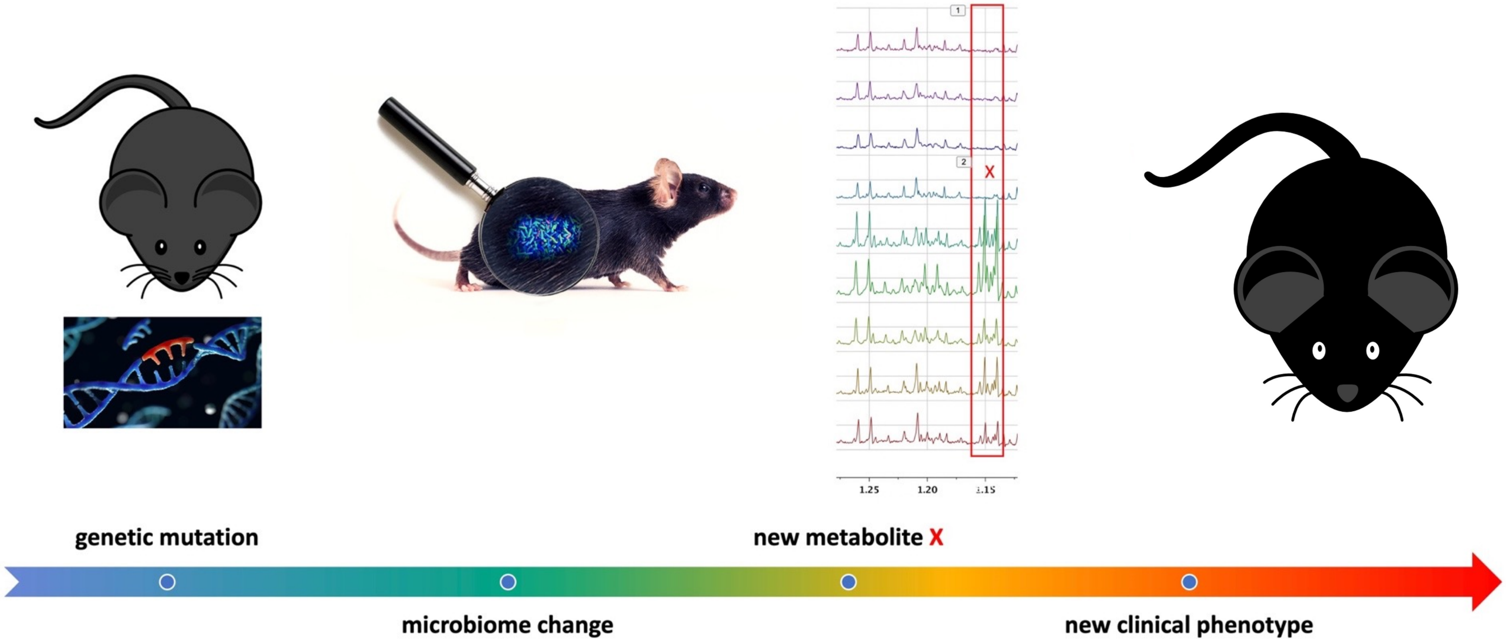

Supplement: Supplementary file 2 [file DataSheet1.PDF]
